# Ubiquitin modification in osteogenic differentiation and bone formation: From mechanisms to clinical significance

**DOI:** 10.3389/fcell.2022.1033223

**Published:** 2022-10-21

**Authors:** Yuan Pan, Yiman Tang, Hang Gu, Wenshu Ge

**Affiliations:** ^1^ Department of General Dentistry II, Peking University School and Hospital of Stomatology, National Center of Stomatology, National Clinical Research Center for Oral Diseases, National Engineering Research Center of Oral Biomaterials and Digital Medical Devices, Beijing, China; ^2^ Fourth Clinical Division, Peking University School and Hospital of Stomatology, National Center of Stomatology, National Clinical Research Center for Oral Diseases, National Engineering Research Center of Oral Biomaterials and Digital Medical Devices, Beijing, China

**Keywords:** ubiquitin modification, epigenetics, mesenchymal stem cells, osteogenic differentiation, protein deubiquitinases

## Abstract

The ubiquitin–proteasome system is an important pathway for mediating posttranslational modification and protein homeostasis and exerts a wide range of functions in diverse biological processes, including stem cell differentiation, DNA repair, and cell cycle regulation. Many studies have shown that ubiquitination modification plays a critical role in regulating the osteogenic differentiation of stem cells and bone formation through various mechanisms. This review summarizes current progress on the effects and mechanisms of ubiquitin modification on transcription factors and signaling pathways involved in osteogenic differentiation. Moreover, the review highlights the latest advances in the clinical application of drugs in bone tissue engineering. A thorough understanding of ubiquitin modifications may provide promising therapeutic targets for stem cell-based bone tissue engineering.

## 1 Introduction

The reconstruction of large bone defects caused by trauma, infection, bone tumor resection, etc., is challenging due to the limited self-repair ability of bone tissue. In recent years, the development of bone tissue engineering has increased the efficiency of bone reconstruction. Mesenchymal stem cells (MSCs) are considered the most promising, because these adult stem cells possess the ability to self-renew and undergo differentiation into diverse cell lineages, such as osteoblasts, adipocytes, and chondrocytes (Squillaro et al., 2016; Lin et al., 2019). However, the molecular mechanism of osteogenic differentiation of MSCs remains unclear, posing a barrier to the further development of MSC-based treatment for bone defects. Thus, studies on the regulation of the osteogenic differentiation of MSCs will help identify new targets.

Cell differentiation is determined by genetic and epigenetic mechanisms (Huang et al., 2015). Epigenetic modifications are genetic alterations in gene function without affecting DNA sequence and include DNA methylation, histone modification, and regulation by noncoding RNA (Egger et al., 2004). Recent studies have shown that stemness regulatory factors in conjunction with chromatin remodeling complexes, histone modification, or posttranslational modification of chromatin binding factors play an important role in stem cell fate determination (Orkin and Hochedlinger, 2011; Atlasi and Stunnenberg, 2017). As one of the important protein posttranslational modifications, ubiquitination dynamically regulates protein stability, protein localization, and signal transduction, and thus, affects cell cycle proliferation, apoptosis, and differentiation (Reyes-Turcu and Wilkinson, 2009; Frezza et al., 2011). Ubiquitination is reversible, and deubiquitination is achieved by deubiquitinating enzymes (Suresh et al., 2016). Ubiquitin modification regulates not only proteasome-mediated degradation but also biological processes, such as DNA repair, endocytosis, autophagy, transcription, immunity, and inflammation (Kwon et al., 2017). Understanding ubiquitin modification in the osteogenic differentiation of stem cells is of great clinical significance in orthopedic disease and would advance the development of MSC-based bone tissue engineering.

This review summarizes the role and mechanism of ubiquitin modification in the osteogenic differentiation of stem cells and discusses the latest progress in preclinical studies and future applications. Moreover, this review highlights the recently completed or ongoing clinical trials and discusses the major challenges and use of ubiquitin modification in therapeutic application.

## 2 Methods

A primary database was established for all relevant articles (February 2001–September 2022) based on the PubMed database. The following keywords and their combinations were used: (ubiquitin OR ubiquitination [Title/Abstract] OR deubiquitin [Title/Abstract] OR deubiquitination [Title/Abstract]) AND (osteogenesis [Title/Abstract] OR osteogenic [Title/Abstract]). Titles and abstracts were selected based on the following criteria:

1) Only original research articles were included.
2) Studies based on ubiquitination in the osteogenic differentiation of MSCs, including the ubiquitin–proteasome system (UPS), ubiquitin ligases, and protein deubiquitinating enzymes, were included.

**FIGURE 1:**
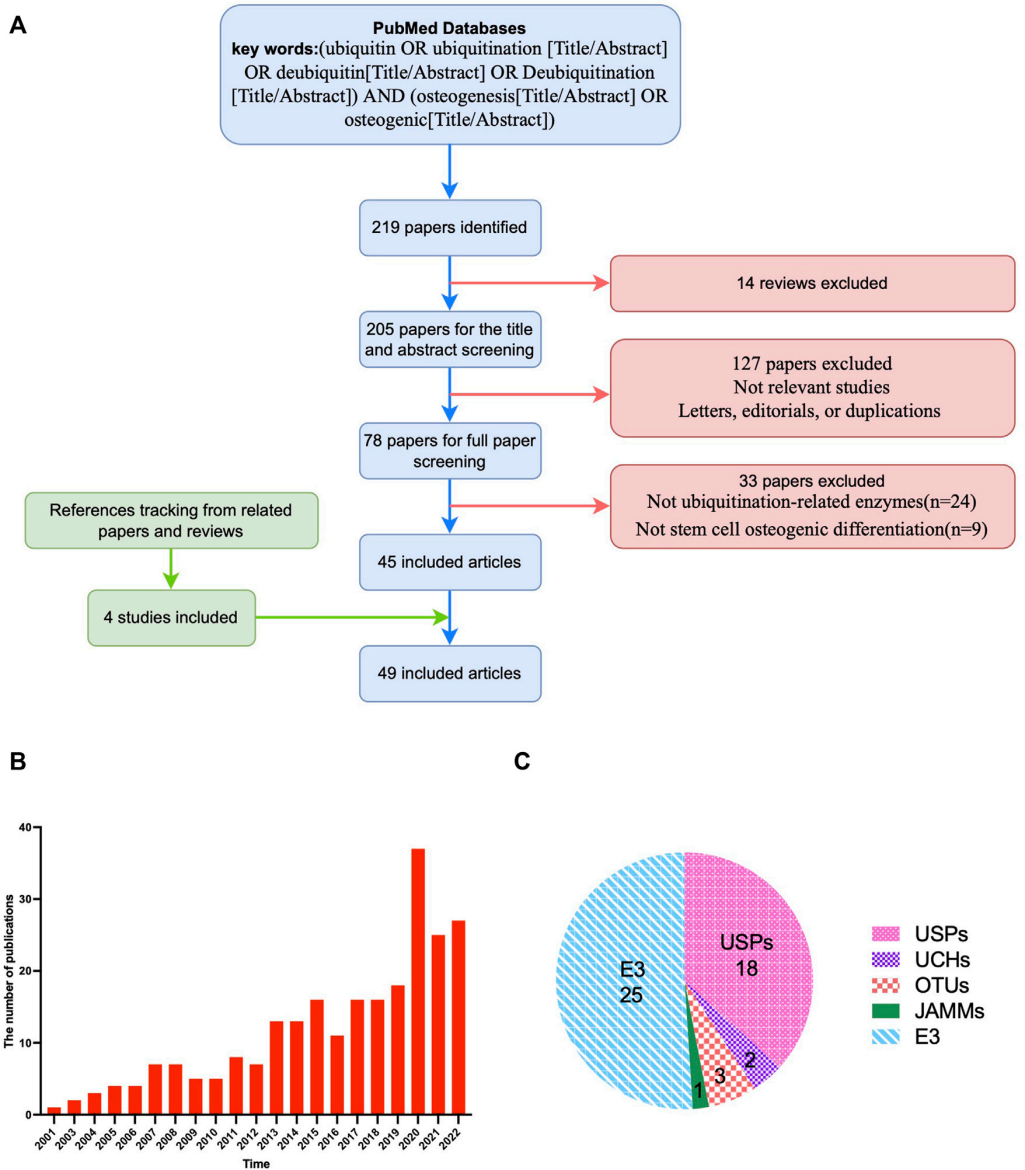
Overview of the included articles for the role and mechanism of ubiquitin modification in osteogenic differentiation of stem cells. **(A)**, Flow diagram illustrating the study screening and inclusion process. **(B)**, Statistics for the numbers of publications in different years. **(C)**, Types of enzymes of the included articles. The number refers to the number of relevant literatures. USPs, ubiquitin-specific proteases; UCHs, Ubiquitin C-Terminal Hydrolase; OTUs, ovarian tumor-related proteases; MJDs, Machado-Josephin domain proteases; JAMMs, Jabl/MPN domain associated metalloisopeptidase; E3, ubiquitin ligases.

A total of 219 articles were retrieved after the initial database search, and then 14 reviews were excluded. The titles and abstracts were screened, and 123 articles were excluded because they were not relevant to the current analysis or included letters, editorials, or duplicate reports. Of the 82 potentially relevant studies, 37 were further excluded after the full-text was reviewed, because 29 were not related to ubiquitination-related enzymes and 8 were not related to stem cell osteogenic differentiation. Reference tracking was performed on the full-texts of the resulting articles to identify missing articles that met the inclusion criteria. Four articles fulfilled the inclusion criteria. The final number of included articles was 49 (Figure 1A). The number of articles in this field has steadily increased over the last 2 decades, demonstrating the importance of ubiquitination modification in bone tissue engineering (Figure 1B). Among the included studies, ubiquitin-specific protease (USP) and ubiquitin ligase were the most frequently investigated enzymes that affect stem cell osteogenic differentiation, accounting for more than three-quarters of the total (Figure 1C).

## 3 Ubiquitinating and deubiquitinating enzymes

Ubiquitin—a highly conserved protein composed of 76 amino acids—binds a protein substrate as a monomer or polymer (Komander and Rape, 2012; Kulathu and Komander, 2012). Ubiquitin contains seven lysine (K) residues: K6, K11, K27, K29, K33, K48, and K63, each of which can form isopeptide bonds with the COOH terminus of other ubiquitin molecules, thereby constituting a polyubiquitin chain. Polyubiquitin chains formed by K48 and K63 chains are the most prevalent ubiquitin chains. Polyubiquitination through K48 guides protein degradation by the 26S proteasome, whereas that through K63 regulates signal transduction and protein activity (Chandrasekaran et al., 2017). In eukaryotes, UPS is one of the most important protein degradation pathways, with high selectivity. UPS mainly degrades misfolded proteins, short-lived proteins, proteins that must be cleared in response to signal-based stimulation, and proteins that must be rapidly degraded by transformation from long-lived proteins. UPS primarily relies on three ubiquitin-related enzymes and protein deubiquitinating enzymes.

Ubiquitination is a three-enzyme cascade reaction involving three enzymes: E1 ubiquitin-activating enzyme, E2 ubiquitin-conjugating enzyme, and E3 ubiquitin ligating enzyme. First, E1 activates ubiquitin by linking the C-terminal of ubiquitin to a cysteine residue in E1 through ATP-dependent thioester bond formation. Second, E1 transfers activated ubiquitin to E2. Last, E3 catalyzes ubiquitin transfer from E2 to lysine residues on target proteins. E3 can identify target proteins that must be ubiquitinated; therefore, the last step occurs in a subunit-specific manner and is highly regulated (Foot et al., 2017). Multiple ubiquitination events lead to the formation of polyubiquitin chains whose lysine or amino terminus can be used to polymerize ubiquitin, thereby amplifying the polyubiquitin signal. Different polyubiquitin chains are associated with varied cellular outcomes (Mevissen and Komander, 2017). Receptors recognize diverse ubiquitin modifications linked to target proteins, resulting in different signaling outputs. These receptors have ubiquitin-binding domains that interact with ubiquitin or polyubiquitin, and may also have domains that interact with modified target proteins or other macromolecules.

As with most posttranslational modifications, ubiquitination is reversible. Deubiquitination is accomplished by enzymes collectively known as protein deubiquitinases (DUBs). DUBs are a large family of proteases comprising the catalytic domain, ubiquitin-interacting motif, ubiquitin-associated domain, ubiquitin-binding domain, ubiquitin-like folding domain, and zinc-finger USP domain. These domains facilitate the binding and recognition of different ubiquitin chains. The human genome encodes nearly 100 DUBs, making it the largest family of UPS. DUBs are classified into two major groups: the cysteine protease family and the metalloprotease family (Table 1). (Guo et al., 2018) The enzymatic activity of cysteine proteases is dependent on the sulfhydryl group at the active site and deprotonation of cysteine is assisted by the adjacent histidine, polarized by aspartic acid residues. This family contains USP, ubiquitin C-terminal hydrolases (UCHs), Machado–Josephin domain proteases, and ovarian tumor-related proteases (OTUs). However, the metalloprotease family only contains Jab1/MPN domain-associated metalloisopeptidases (JAMMs), which use Zn2+-bonded polarized water molecules to form noncovalent intermediates with substrates. Metal atoms are mainly stabilized by one aspartic acid and two histidine residues, and the intermediates are further hydrolyzed by proton transfer from water molecules, thus releasing DUBs (Rahman et al., 2015; Mevissen and Komander, 2017).

**TABLE 1:** Members of protein deubiquitination enzymes.

Group	Family	Members
cysteine protease	USPs	USPL1, CYLD, USP1, USP2, USP3, USP4, USP5, USP6, USP7, USP8, USP9x, USP10, USP11, USP12, USP13, USP14, USP15, USP16, USP17L2, USP18, USP19, USP20, USP21, USP22, USP23, USP24, USP25, USP26, USP27, USP28, USP29, USP30, USP31, USP32, USP33, USP34, USP35, USP36, USP37, USP38, USP39, USP40, USP41, USP42, USP43, USP44, USP45, USP46, USP47, USP48, USP49, USP50, USP51, USP52, USP53, USP54
	UCHs	UCH-L1, UCH-L3, UCH37/UCH-L5, BAP1
	MJDs	ATXN3, ATXN3L, JOSD1, JOSD2
	OTUs	OTUB1, OTUB2, OTUD4, OTUD5, OTUD6A, OTUD6B, OTU1, HIN1L, A20, Cezanne, Cezanne2, TRABID, VCPIP1
metalloprotease	JAMMs	BRCC36, CSNS, POH1, AMSH, AMSH-LP, MPND, MYSM1, PRPF8

DUBs hydrolyze ubiquitin molecules on target proteins by hydrolyzing ester, peptide, or isopeptide bonds at the carboxyl terminal of ubiquitin. Additionally, DUBs can process ubiquitin precursors and degrade unbound ubiquitin synthesized *de novo* or released by other DUBs. Thus, they identify distinct forms of ubiquitin and polyubiquitin, similar to cell-targeted receptors. Deubiquitination is involved in many cellular functions, including DNA repair, protein degradation, cell cycle regulation, stem cell differentiation, and cell signaling. DUBs play a key role in bone tissue engineering by regulating stem cell differentiation and function (Guo et al., 2018).

## 4 Mechanism of ubiquitin modification regulating the osteogenic differentiation of stem cells

**FIGURE 2:**
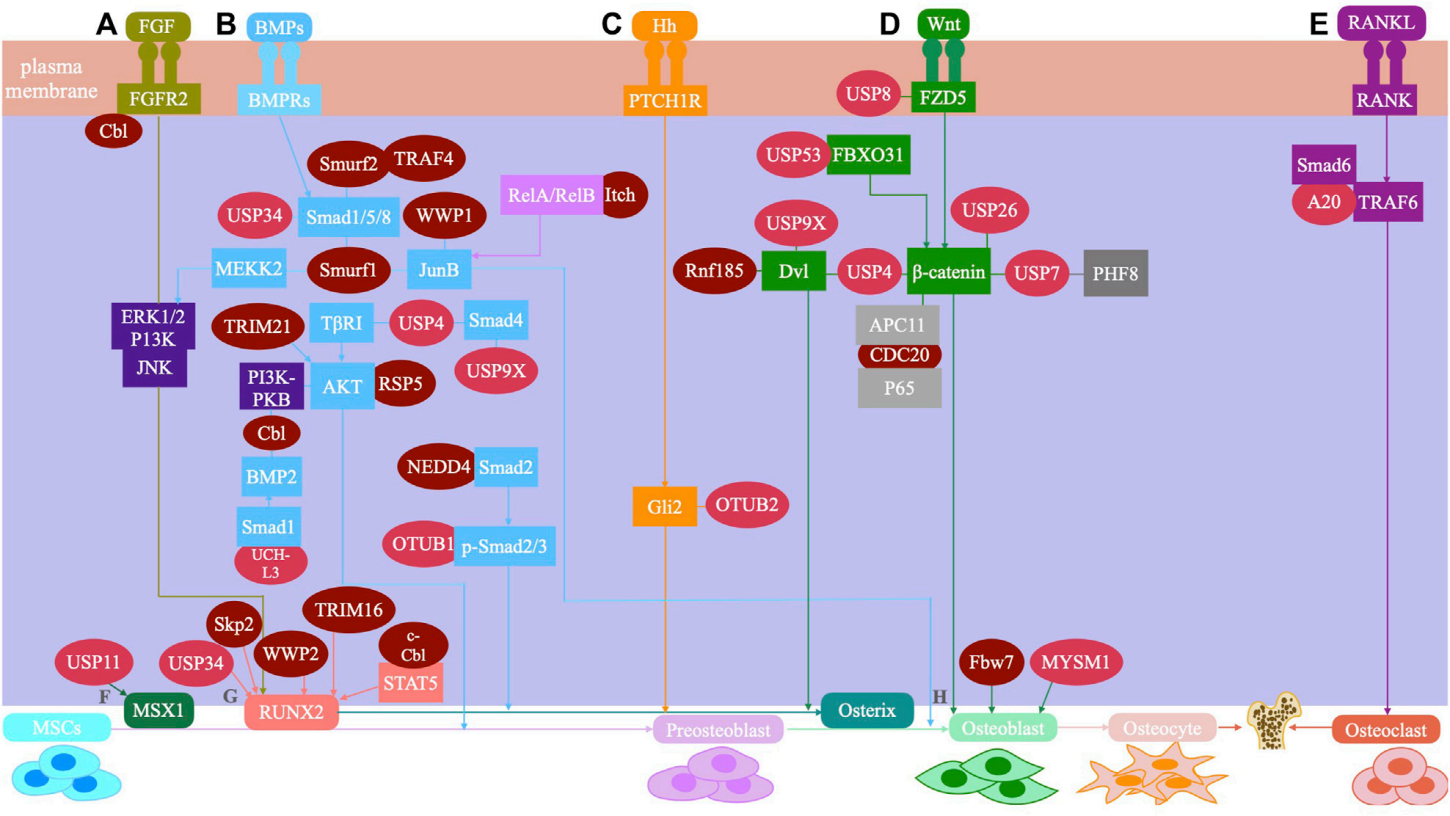
The UPS system mediated signaling pathway and regulatory factors during osteogenic differentiation of mesenchymal stem cells are shown. **(A)** FGF signaling: Smurfl inhibits JNK signaling by promoting MEKK2 ubiquitination and degradation, and negatively regulates FGF signaling. Cbl can reduce the ubiquitination of PDGFR and FGFR2, thus inhibiting FGF signaling. **(B)** BMP signaling: USP4 can deubiquitinate TI3RI and Smad4, and enhance BMP signaling. USP9X can antagonize Smad4 monoubiquitination and enhance BMP signaling. USP34 can activate Smad1/5/8 to enhance BMP signaling. UCH-L3 can deubiquitinate and stabilize Smadl, and enhance BMP signaling. OTUB1 deubiquitinates the p-Smad2/3 complex and enhances BMP signaling. Smurfl and Smurf2 induce Smad1/5/8 ubiquitination and negatively regulates BMP signaling. Smurfl and WWP1 promote ubiquitination and degradation of JunB and inhibit BMP signaling. Itch enhances JunB by ubiquitinating ReIA/Re1B and positively regulates BMP signaling. RSP5 induces ubiquitination of PKB/Akt and positively regulates BMP signaling. TRIM21 accelerated the degradation of Akt to enhance BMP signaling. Cbl reduces BMP signaling by inhibiting PI3K-PKB/Akt pathway and ubiquiting BMP2. TRAF4 positively regulates BMP signaling by ubiquitinating Smurf2. **(C)** Hedgehog signaling: OTUB2 enhances Hh signaling through deubiquitinating Gli2. **(D)** Wnt signaling: USP4 enhances Wnt signaling through deubiquitinating and stabilizing P-catenin. Conversely, USP4 inhibits Wnt signaling by deubiquitinating Dvl. USP9X enhances Wnt signaling through deubiquitinating Dv12. USP8 enhances Wnt signaling through deubiquitinating FZD5. USP53 inhibits proteasome degradation of p-catenin by interacting with FBXO31 and enhance Wnt signaling. USP7 can activate Wnt signaling through deubiquitinating P-catenin. USP26 can activate Wnt signaling by stabilizing p-catenin. Rnf185 inhibits the Wnt signaling by promoting ubiquitin and degradation of Dv12. CDC20 enhances Wnt signaling by promoting P65 degradation in an APC11-dependent manner. **(E)** Sonic hedgehog (Shh) signaling: A20 inhibits polyubiquitination of TRAF6. **(F)** MSX1: USP11 can deubiquitinate the MSX1 protein. **(G)** RUNX2: USP34 can stabilize RUNX2. WWP2 catalyzes the monoubiquitination of RUNX2. c-Cbl mediates STAT5 ubiquitination and inhibits RUNX2. Skp2 targets Runx2 degradation. TRIM16 stabilizes RUNX2 protein levels. **(H)** Osteoblast: MYSM1 can affect the maturation and differentiation of osteoblasts. Fbw7 negatively regulates Osx protein stability and osteoblast differentiation.

Ubiquitin modification regulates many signaling pathways and transcription factors during the osteogenic differentiation of stem cells. The major signaling pathways involve transforming growth factor β (TGF-β)/bone morphogenetic protein (BMP), Wnt/β-catenin, hedgehog, fibroblast growth factor, nuclear factor kappa-B (NF-κB), and parathyroid hormone signaling pathways (Rahman et al., 2015; Fan et al., 2017; Sun et al., 2018). Additionally, ubiquitin modification also plays a vital role in the osteogenic differentiation of stem cells by regulating transcription factors, such as runt-related transcription factor 2 (RUNX2). (Lim et al., 2016) (Figure 2).

### 4.1 Ubiquitin ligase E3

E3 includes the homologous to E6-APC terminus (HECT) domain, RING domain, and U-box domain families of ubiquitin ligases. Some studies have implicated E3 ubiquitin ligases in the regulation of osteogenic differentiation in stem cells. (Shen et al., 2021).

#### 4.1.1 The HECT domain family

The neural precursor cell-expressed developmentally downregulated gene 4 (Nedd4), belonging to the HECT domain family, is one of the most studied and understood E3 enzymes. Nedd4 includes Smad ubiquitination regulatory factor 1 (Smurf1), Smurf2, WW domain-containing E3 ubiquitin protein ligase 1 (WWP1), WWP2, atrophin-1-interacting protein 4 (or Itch), Nedd4, Nedd4-like (Nedd4L) E3 ubiquitin protein ligase, Nedd4-like ubiquitin protein ligase 1 (NEDL1), and NEDL2. Smurf1—the first ubiquitin ligase implicated in bone formation—regulates osteoblast metabolism by ubiquitinating TGF superfamily proteins Smad (Smad1 and Smad5) and RUNX2 (Ying et al., 2003). Moreover, Smurf1 was reported to block osteoblast differentiation in various ways. For example, Smurf1 inhibits the c-Jun N-terminal kinase signaling pathway by promoting MAP kinase kinase-2 ubiquitination and degradation, thereby inhibiting osteoblast differentiation (Yamashita et al., 2005). Smurf1 was reported to inhibit osteoblast differentiation by promoting the degradation of the AP-1 family transcription factor JunB (Zhao et al., 2010). Smurf2 induces Smad1/5 ubiquitination and negatively regulates BMP/Smad signaling, thereby inhibiting the osteogenic differentiation of MSCs (Kushioka et al., 2020). Furthermore, WWP1 promotes the ubiquitination and degradation of JunB to inhibit osteoblast differentiation (Zhao et al., 2011). WWP2 positively regulates osteogenesis by catalyzing the monoubiquitination of RUNX2 and promoting the transactivation of RUNX2 (Zhu et al., 2017). Nedd4 was reported to promote bone formation by activating TGFβ1 signaling to increase osteoblast proliferation (Jeon et al., 2018). Itch, which contains the WW domain, was reported to inhibit osteogenic differentiation (Liu et al., 2017). Nedd4L or RSP5, of the HECT domain family, induces K63-linked ubiquitination of protein kinase B (PKB/AKT), promoting the osteogenic differentiation of MSCs through the AKT signaling pathway (Liang et al., 2020).

#### 4.1.2 Casitas B-lineage lymphoma family

Casitas B-lineage lymphoma (Cbl), an E3 ligase, was reported to negatively regulate osteogenic differentiation. Cbl can reduce the stability and nuclear localization of osterix by inhibiting the phosphoinositide-3-kinase–PKB/AKT pathway, thus inhibiting osteogenic differentiation (Scanlon et al., 2017). According to a study, Cbl reduces the ubiquitination of platelet-derived growth factor receptor and fibroblast growth factor receptor 2 by inhibiting their interaction with receptor tyrosine kinases, thus indicating osteogenic differentiation of MSCs (Severe et al., 2011). Cbl-b and c-Cbl, members of the Cbl family, suppress the osteogenic differentiation of BMP2-induced MSCs and play essential roles in bone formation through an increase in UPS-mediated osterix degradation (Choi et al., 2015). According to a detailed study, c-Cbl mediated the ubiquitination of signal transducers and activators of transcription 5 protein and reduced the expression of insulin-like growth factor 1, thereby inhibiting the osteogenic differentiation of MSCs (Dieudonne et al., 2013).

#### 4.1.3 The U-box domain family

S-phase kinase associated protein 2 of the Skp–Cullin–F-box family negatively regulates osteogenic differentiation by targeting RUNX2 degradation (Thacker et al., 2016). F-box/WD repeat-containing protein 7, another component of the Skp–Cullin–F-box ubiquitin ligase complex, has been shown to negatively regulate osterix protein stability and osteoblast differentiation (Hoshikawa et al., 2020).

#### 4.1.4 Other E3 ligases

In addition to the well-known ubiquitin ligases discussed, other ubiquitin ligases have been implicated in the regulation of osteogenic differentiation. Ring finger protein 185 inhibits the WNT signaling pathway by promoting the ubiquitination and degradation of Dvl2, thus negatively regulating osteogenic differentiation (Zhou et al., 2014). Tripartite motif (TRIM) 16 was found to stabilize RUNX2 by reducing the CHIP-mediated ubiquitination and degradation of K48-linked RUNX2, thereby promoting the osteogenic differentiation of human periodontal ligament stem cells (Zhao et al., 2020). TRIM21 was reported to promote the K48-linked ubiquitination of AKT, which accelerated the degradation of AKT to inhibit the osteotropic signaling pathway, thus inhibiting the osteogenic differentiation of MSCs (Xian et al., 2022). The cell division cycle 20 protein promotes P65 degradation in an anaphase-promoting complex subunit 11-dependent manner, which in turn promotes osteogenic differentiation (Du et al., 2021). The ubiquitin ligase TNF receptor-associated factor (TRAF)4 positively regulates the osteogenic differentiation of MSCs by mediating the K48-linked ubiquitination and degradation of Smurf2 at K119 (Li et al., 2019). The roles and mechanisms of E3 ubiquitin ligases on the osteogenic differentiation of stem cells are summarized in Table 2.

**TABLE 2:** The mechanism and effect of E3 on osteogenic differentiation of MSCs.

Enzyme	Pathway	Substrate	Function	Author, year, reference
Smurf1	RUNX2		Inhibit	Ying et al. (2003)
	TGF-β/BMP	Smad5	Inhibit	Ying et al. (2003)
	JNK	MEKK	Inhibit	Yamashita et al. (2005)
	JunB		Inhibit	Zhao et al. (2010)
Smurf2	TGF-β/BMP	Smad1/5	Inhibit	Kushioka et al. (2020)
WWP1	TNF	JunB	Inhibit	Zhao et al. (2011)
WWP2	RUNX2		Promote	Zhu et al. (2017)
NEDD4	TGF-β	Smad1/2	Promote	Jeon et al. (2018)
Itch	NF-κB	RelA/RelB	Inhibit	Liu et al. (2017)
RSP5	AKT	PKB	Promote	Liang et al. (2020)
Cbl	Osterix	P13K/AKT	Inhibit	Scanlon et al. (2017)
	ERK1/2 ,P13K	PDGFR, FGFR2	Inhibit	Severe et al. (2011)
	BMP2		Inhibit	Choi et al. (2015)
	RUNX2	STAT5	Inhibit	Dieudonne et al. (2013)
Skp2	RUNX2		Inhibit	Thacker et al. (2016)
Fbw7	Osx		Inhibit	Hoshikawa et al. (2020)
Rnf185	Wnt/β-catenin	Dvl2	Inhibit	Zhou et al. (2014)
TRIM16	RUNX2	Chip	Promote	Zhao et al. (2020)
TRIM21	AKT		Inhibit	Xian et al. (2022)
CDC20	p65	APC11	Promote	Du et al. (2021)
TRAF4	Smurf2		Promote	Li et al. (2019)

### 4.2 DUBs

#### 4.2.1 The USP family

The USP family is the largest and most diverse family of DUBs, with 56 members in humans. The catalytic domain of USP is composed of six conserved motifs, of which two are named Cys- and His-box. (Guo et al., 2018).

USP4, USP11, and USP15 share highly similar domains and protein sequences. TGF-β/BMP signaling plays a key role in the osteogenic differentiation of MSCs and bone formation. USP4, USP11, and USP15 act on type I TGF-β receptors (TβRI) (Garg et al., 2017). USP4 can enhance TGF-β signaling by directly interacting with TβRI/ALK5. USP4 is phosphorylated by AKT and translocated to the cytoplasm and membrane, where USP4 binds TβRI to deubiquitinate and protect it from damage (Zhang et al., 2012). Moreover, USP4 stabilizes Smad4 and activates BMP signaling by inhibiting Smad4 monoubiquitination (Zhou et al., 2017). According to a study, USP4 activates Wnt signaling by deubiquitinating and stabilizing β-catenin (Yun et al., 2015). By contrast, USP4 was reported to inhibit Wnt signaling by deubiquitinating the polyubiquitin chains on dishevelled (Dvl/Dsh) and blocking Wnt3a-induced osteoblast differentiation and bone formation (Zhou et al., 2016). Additionally, USP11 promotes the osteogenic differentiation of MSCs by interacting and deubiquitinating Msh homeobox 1 (Kaushal et al., 2022).

USP9X deubiquitinates Smad4 at K519, which inhibits transcription by preventing its binding to phosphorylated Smad2/3. Thus, USP9X activates TGF-β signaling by antagonizing Smad4 monoubiquitination (Dupont et al., 2009). In addition, USP9X-mediated deubiquitination of dishevelled protein 2 (Dvl2) is necessary for canonical Wnt activation (Nielsen et al., 2019). A study has shown that USP34 promotes osteogenic differentiation by activating Smad1/5/8 and stabilizing the transcription factor RUNX2 (Guo et al., 2020).

USP8 promotes osteogenic differentiation by blocking the ubiquitination of Wnt receptor frizzled 5 (FZD5) to stabilize the Wnt/β-catenin signaling pathway (Chaugule et al., 2021). USP53 inhibits proteasome degradation of β-catenin by interacting with F-box only protein 31 (FBXO31), thereby promoting osteogenic differentiation of human bone marrow-derived MSCs (Baek et al., 2021). According to Tang, USP7 can promote the osteogenic differentiation of MSCs through its deubiquitinating activity (Tang et al., 2017). Moreover, USP7 was reported to activate the Wnt/β-catenin signaling pathway by interacting directly with β-catenin (Zeng et al., 2019). USP26 promotes the osteogenic differentiation of MSCs by stabilizing β-catenin and impairs the osteoclastic differentiation of bone myelomonocytes (BMMs) by stabilizing inhibitors of NF-κBα (Li et al., 2022).

Studies on USP42 suggest that USP42 regulates transcription by binding and deubiquitinating histone H2B (Hock et al., 2014). USP42 was reported to negatively regulate Wnt/β-catenin signaling and promote the clearance of the Wnt receptor (Giebel et al., 2021), suggesting that USP42 is involved in the regulation of stem cell differentiation.

#### 4.2.2 The UCH family

The UCH family members contain an N-terminal catalytic domain of 230 residues, frequently followed by C-terminal extensions that mediate protein–protein interactions. The four human UCHs are divided into smaller UCHs (UCH-L1 and UCH-L3) and larger UCHs (UCH37/UCH-L5, and BAP1) based on substrate specificity (Eletr and Wilkinson, 2014). UCH-L3 activates osteoblast differentiation by deubiquitinating and stabilizing Smad1 (Kim et al., 2011). Moreover, UCH-L3 was reported to activate NF-κB signaling by deubiquitinating and stabilizing the ubiquitin ligase TRAF2 (Zhang et al., 2020).

#### 4.2.3 The OUT family

The OTU family is identified according to their homology with ovarian tumor genes. A total of 15 genes in the human genome are grouped into three subclasses: OTUB, OTU, and A20-like OTU (Eletr and Wilkinson, 2014). A20 recruited by Smad6 inhibits the TGF-β1-induced, K63-linked polyubiquitination of TRAF6 and contributes to the negative regulation of nonstandard TGF-β signaling (Jung et al., 2013), suggesting that A20 regulates osteogenic differentiation through this primary pathway. OTUB1 inhibits the ubiquitination of phospho-SMAD2/3 by binding to and inhibiting E2, independent of its catalytic activity, thereby activating TGF-β signaling (Herhaus et al., 2013). Other studies have shown that OTUB2 regulates hedgehog signaling by inhibiting the degradation of transcription factor Gli2, thereby promoting osteogenic differentiation (Li et al., 2018).

#### 4.2.4 Machado-Josephin domain proteases MJDs

The Machado–Josephin domain proteases family has four members, which contain the catalytic triad domains formed by two highly conserved histidine boxes and one cysteine box (Eletr and Wilkinson, 2014). However, studies on the relationship between Josephin-Dubs and the osteogenic differentiation of stem cells are lacking.

#### 4.2.5 JAMM domain proteins

Humans possess eight JAMMs domain proteins in humans (Eletr and Wilkinson, 2014). MYSM1, a member of the JAMMs family, has been shown to affect the maturation and differentiation of osteoblasts (Haffner-Luntzer et al., 2018); however, its effect on osteogenic differentiation of stem cells has not been reported.

Table 3 summarizes the mechanisms and effects of DUBs on the osteogenic differentiation of MSCs.

**TABLE 3:** The mechanism and effect of DUBs on osteogenic differentiation of MSCs.

Enzyme	Pathway	Substrate	Function	Author, year, reference
USP4	TGF-β/BMP	ALK5	Promote	Garg et al. (2017)
				Zhang et al. (2012)
		Smad4	Promote	Zhou et al. (2017)
	Wnt/β-catenin	β-catenin	Promote	Yun et al. (2015)
		Dvl/Dsh	Inhibit	Zhou et al. (2016)
USP11	MSX1		Promote	Kaushal et al. (2022)
USP9x	TGF-β/BMP	Smad4	Promote	Dupont et al. (2009)
	Wnt/β-catenin	Dvl2	Promote	Nielsen et al. (2019)
USP34	TGF-β/BMP	Smad1/5/8	Promote	Guo et al. (2020)
USP8	Wnt/β-catenin	FZD5	Promote	Chaugule et al. (2021)
USP53	Wnt/β-catenin	FBXO31	Promote	Baek et al. (2021)
USP7	Wnt/β-catenin	β-catenin	Promote	Zeng et al. (2019)
USP26	Wnt/β-catenin	β-catenin	Promote	Li et al. (2022)
USP42	Wnt/β-catenin	ZNRF3/RNF43	Inhibit	Hock et al. (2014)
		H2B	Inhibit	Giebel et al. (2021)
UCH-L3	TGF-β/BMP	Smad1	Promote	Kim et al. (2011)
	NF-κB	TRAF2	Promote	Zhang et al. (2020)
A20	TGF-β	TRAF6	Inhibit	Jung et al. (2013)
OTUB1	TGF-β	p-Smad2/3	Promote	Herhaus et al. (2013)
OTUB2	Hh	Gli2	Promote	Li et al. (2018)
MYSM1			Promote	Haffner-Luntzer et al. (2018)

Ubiquitination and deubiquitination regulate osteogenic differentiation of stem cells through multiple pathways. If specific ubiquitin-related enzymes that regulate osteogenic differentiation can be systematically screened out, it is expected to participate in the regulation of stem cells fate through overexpression or inhibition of a series of enzymes. Therefore, mapping the interaction between ubiquitination and deubiquitination in the stem cell regulatory network may have a significant impact on tissue engineering and translational medicine research.

## 5 Application of ubiquitin modification in bone tissue engineering

In recent years, the use of UPS as a new target for disease treatment has gradually attracted people’s attention. Bortezomib was the first proteasome inhibitor to be approved for use in the treatment of multiple myeloma (Oyajobi et al., 2007). Bortezomib is believed to treat postmenopausal osteoporosis by suppressing Smurf-mediated SMAD ubiquitination (Fang et al., 2021). In clinical studies, bortezomib was reported to increase the proliferation and differentiation of osteoblasts by increasing β-catenin levels (Qiang et al., 2009). Treatment of mice with low doses of bortezomib (approximately one-fifth to one-third the dose equivalent required for the antitumor effect) increased bone formation and mineralized trabecular bone (Mukherjee et al., 2008). This strategy may be used to increase bone volume in malignant osteolytic disease or osteoporosis. Another proteasome inhibitor, withaferin A, was reported to promote fracture healing and regulate bone anabolism in osteoporosis (Khedgikar et al., 2013). In addition, local injection of the proteasome inhibitor PS1 and cyclooxygenin promoted fracture healing in rats (Yoshii et al., 2015). Melatonin is a type of indolamine with many biological functions. Melatonin was reported to promote bone formation through the BMP/MAPK/Wnt signaling pathway (Luchetti et al., 2014). Moreover, melatonin reversed TNFα-mediated inhibition of osteogenesis in MSCs by stabilizing Smad1 and is a promising therapeutic agent for TNFα-mediated inhibition of osteogenesis (Lian et al., 2016).

The developments in tissue engineering have made stem cell transplantation feasible for treating bone-related diseases, such as osteoporosis. In the transplantation process, pretreatment of cells with ubiquitin-related enzymes and their small molecule inhibitors increases osteogenic differentiation. The proteasome inhibitor lactacystin accelerated BMP-induced osteogenic differentiation by increasing the levels of phosphorylated Smad proteins. Optimized lactacystin treatment in a clinical setting may facilitate autogenous or BMP-induced bone formation in bones with defects (Ito et al., 2011). In addition, osteocytes also play an important role in bone remodeling (Currey et al., 2017). Osteocytes produce proteins, including receptor activator of NF-κB ligand (RANKL) and sclerostin, which contribute to both bone resorption and formation. RANKL is essential for osteoclast formation, and sclerostin inhibits canonical Wnt signaling required for osteoblast differentiation (Li et al., 2005; Xiong et al., 2015). Damaged osteocytes repair themselves through autophagy. Some evidence suggests that inhibiting autophagy in osteocytes promotes osteocyte death as well as some aging-associated bone changes. However, if osteocyte injury is severe, autophagy cannot repair the damage, and osteocyte apoptosis is induced. Local osteocyte apoptosis increases RANKL levels in surrounding osteocytes, which results in an increase in osteoclasts (Jilka and O'Brien, 2016). A study showed that casein kinase 2-induced phosphorylation of USP4 stabilizes sirtuin1 by inhibiting ubiquitin-dependent proteasomal degradation. Upregulation of sirtuin1 inhibited sclerostin transcription in osteocytes, which is essential for maintaining bone homeostasis (Kim et al., 2022). Another study reported that USP10 could maintain the stability of p53. In addition, estrogen can induce p53 degradation by regulating USP10 in osteocytes, thereby preventing cellular senescence and bone loss (Wei et al., 2021). Therefore, cell therapy targeting USP4 or USP10 may repair damaged osteocytes and thus, achieve bone regeneration.

Recent animal-based studies have reported the significance of ubiquitin modification-related enzymes and their inhibitors or activators in bone tissue engineering. The inhibitor of USP7 activity HBX 41108 blocks the osteogenic differentiation of hMSCs (Tang et al., 2017). In a study, the loss of USP34 in MSCs impaired fixation of mouse titanium implants. Thus, USP34 is an important drug target for promoting the osteogenic differentiation of stem cells (Xue et al., 2020). RTA-408, an activator of nuclear factor-erythroid 2-related factor 2, was reported to inhibit osteoclast formation *in vitro*. In this study, the researchers established a model of oophorectomy-induced osteoporosis, demonstrating that RTA-408 inhibited NF-κB signaling by suppressing the recruitment of TRAF6 to stimulator of interferon genes protein (Sun et al., 2020). Another study demonstrated that oltipraz, an activator of nuclear factor-erythroid 2-related factor 2, rescued the osteoporotic phenotype induced by 1,25(OH)2D deficiency in male mice. Thus, oltipraz can potentially be used for the clinical prevention and treatment of age-related osteoporosis (Yang et al., 2022).

Although several studies have proved the involvement of ubiquitin modification in osteogenic differentiation and bone formation, the clinical use of drugs is limited due to drawbacks, such as low specificity and undesirable dose-dependent side-effects. To overcome the dose-dependent side-effects of high-dose BMP, researchers developed GapmeRs, which are a type of LNA-ASOs with enhanced stability that are clinically safe to modify MSC gene expression, and a non-toxic lipid transport system for Smurf1 silencing. Smurf1 silencing combined with the long-term release of low doses of BMP2 from a biocompatible scaffold promoted bone formation *in vivo*. The findings of this study provide a great possibility for the clinical application of ubiquitination modification in promoting bone formation (Garcia-Garcia et al., 2019).

Many challenges exist in clinically applying the findings of ubiquitination studies. First, many studies have developed small molecule inhibitors that target DUBs; however, screening small molecule compounds has been challenging for pharmaceutical companies due to the unique differences in the three-dimensional structures of the catalytic sites of DUBs. Second, the application of small molecule drugs requires the identification of candidate stem cell populations to improve drug specificity for bone tissue. Simultaneously, the safety of the drug to other tissues must be ascertained. In addition, because the bioavailability of drugs is usually not high, the dose-dependent side-effects of the drugs must be assessed.

## 6 Perspectives and challenges

Because many components of UPS are involved in the regulation of stem cell differentiation, understanding the biochemical roles of ubiquitination modification pathways is critical. Although different family members of UPS have similar catalytic activity centers, they regulate gene expression differentially because of variations in their structure and function. Many types of substrate proteins are targeted by UPS. The enzymes that target transcription factors, cell cycle regulators, and damage repair factors need to be identified and studied. However, studies on regulators of enzyme activity and spatiotemporal specificity during ubiquitination have higher application value than the study of substrates themselves. Therefore, UPS is an attractive drug target. Screening of small molecule inhibitors and developing drugs that target UPS will be a hot topic for future research.

The following research directions are worth discussing. First, new DUBs should be identified, and a gold standard for the characterization of deubiquitinating enzyme should be established. Comparative genomics and proteomics should be used to screen and classify deubiquitinating enzymes. Second, our understanding of UPS function is limited. Therefore, the physiological functions of UPS enzymes, such as their catalytic activity, substrate specificity, and regulation, must be studied to evaluate the critical factors involved in and regulatory networks of UPS. Finally, many studies have shown that the regulation of UPS can bring new ideas for the treatment of various diseases including tumors, but current studies mostly rely on the cellular level and animal level. Systematic and comprehensive clinical studies and verification of the *in vivo* effects of UPS are urgently needed.

In conclusion, ubiquitin modification regulates osteogenic differentiation of stem cells. The development of bone tissue engineering and MSC-based therapies will benefit from a better understanding of the effects and molecular mechanisms of ubiquitination on the osteogenic differentiation of stem cells.
